# Pharmacovigilance of semaglutide: A descriptive analysis of WHO-VigiAccess reports

**DOI:** 10.1097/MD.0000000000048811

**Published:** 2026-05-15

**Authors:** Nasser M. Alorfi, Mansour M. Alourfi, Ammar Aldabbagh, Waleed Saleh Alghamdi, Sarah S. Al Ghamdi, Nasser M. Aldekhail, Mustfa Faisal Alkhanani, Abdulrhman M. Alansari, Faris A. Alsolami, Nihal Abdalla Ibrahim

**Affiliations:** a Pharmacology and Toxicology Department, College of Pharmacy, Umm Al-Qura University, Makkah, Saudi Arabia; b Gastroenterology Department, Dr. Soliman Fakeeh Hospital, Fakeeh Care Group, Jeddah, Saudi Arabia; c Department of Medicine, Division of Gastroenterology, King Abdulaziz University, Jeddah , Saudi Arabia; d Department of Pharmacy, College of Pharmacy, Nursing and Medical Sciences, Riyadh Elm University, Riyadh, Saudi Arabia; e Biology Department, College of Sciences, University of Hafr Al Batin, Hafr Al Batin, Saudi Arabia; f Pharmaceutical Care Services, King Salman Medical City, Madinah Health Cluster, Madinah, Saudi Arabia; g Kholais General Hospital, Makkah Cluster, Ministry of Health, Madinah, Saudi Arabia; h College of Pharmacy, Ajman University, Ajman, United Arab Emirates; i Center of Medical and Bio-Allied Health Sciences Research, Ajman University, Ajman, United Arab Emirates.

**Keywords:** adverse drug reactions, gastroenterology, GLP-1 receptor agonist, pharmacovigilance, semaglutide, WHO-VigiAccess

## Abstract

Semaglutide, a glucagon-like peptide-1 (GLP-1) receptor agonist, is widely prescribed for type 2 diabetes mellitus and chronic weight management. With its growing global use, continuous pharmacovigilance is essential to detect emerging patterns of adverse drug reactions (ADRs). To describe the global ADRs profile of semaglutide using data from the World Health Organization’s (WHO) VigiAccess pharmacovigilance database. A retrospective descriptive analysis was conducted using publicly available ADR data retrieved from the WHO-VigiAccess portal on October 18, 2025. The total number and proportion of ADRs were summarized according to the Medical Dictionary for Regulatory Activities (MedDRA) system organ class (SOC). Demographic information, including age group, sex, geographic region, and reporting year, was reviewed descriptively. A total of 81,770 ADR reports associated with semaglutide were identified. The most frequently reported SOCs were gastrointestinal disorders (28%, n = 42,574), general disorders and administration site conditions (12%, n = 19,200), and injury, poisoning and procedural complications (11%, n = 16,601). Additional categories included nervous system disorders (8%), investigations (7%), and metabolism and nutrition disorders (7%). The majority of ADRs were reported among adults aged 45 to 64 years, with most originating from Europe and the Americas. Annual reporting increased markedly between 2018 and 2025, corresponding with expanded clinical use and obesity-related approvals. Global pharmacovigilance data indicate that semaglutide ADRs are primarily gastrointestinal and systemic in nature, consistent with its known pharmacological effects. Continuous monitoring is warranted to identify emerging safety signals and support optimized patient management as use expands worldwide.

## 1. Introduction

Semaglutide is a long-acting analogue of glucagon-like peptide-1 (GLP-1), a naturally occurring incretin hormone that regulates glucose homeostasis and appetite control.^[1,2]^ By activating GLP-1 receptors, semaglutide enhances glucose-dependent insulin secretion, suppresses glucagon release, slows gastric emptying, and reduces food intake.^[3,4]^ From a gastroenterological perspective, semaglutide exerts significant effects on gastrointestinal physiology through delayed gastric emptying and modulation of gut–brain signaling pathways, which contribute both to its therapeutic benefits and its characteristic gastrointestinal adverse event profile.^[5,6]^ These mechanisms form the pharmacological basis for semaglutide’s therapeutic application in the management of type 2 diabetes mellitus and, more recently, in long-term weight reduction.^[7,8]^ Since its initial approval in 2017, semaglutide has become one of the most widely prescribed GLP-1 receptor agonists, with formulations available for subcutaneous and oral administration.^[9,10]^ Although randomized controlled trials have confirmed its efficacy in glycemic control and cardiovascular risk reduction, real-world experience continues to expand the understanding of its tolerability and safety.^[11–13]^ Gastrointestinal adverse events such as nausea, vomiting, and diarrhea are the most frequently reported treatment-limiting reactions and are well recognized as class effects of GLP-1 receptor agonists.^[14,15]^ However, as clinical indications broaden to include obesity and potential neuroprotective or cardiometabolic uses, a growing number of patients are exposed to semaglutide in diverse therapeutic contexts, raising the importance of continuous post-marketing surveillance.

Semaglutide’s expanding global use and media attention have driven an unprecedented increase in spontaneous adverse drug reaction (ADR reporting, particularly among non-diabetic populations using the drug for weight loss. Understanding these real-world safety patterns is critical for clinicians, policymakers, and researchers to contextualize benefits against potential risks.

The World Health Organization (WHO) Programme for International Drug Monitoring coordinates global pharmacovigilance activities through the VigiBase repository, which collects spontaneous ADR reports from more than 130 member states. Its publicly accessible interface, VigiAccess, enables transparent evaluation of aggregated ADR data classified under the Medical Dictionary for Regulatory Activities (MedDRA). Analyses of VigiAccess data provide valuable insights into worldwide reporting trends, identifying both expected and unexpected reactions that may not be apparent in pre-approval trials. While these data cannot establish causality, they serve as an essential early-signal resource for drug safety assessment and regulatory decision-making.

This study aims to characterize the global ADR profile of semaglutide using WHO-VigiAccess data. By summarizing the distribution of reported ADRs by system organ class (SOC), and examining demographic and geographic trends, the analysis provides a comprehensive overview of semaglutide’s post-marketing safety landscape. The findings contribute to ongoing pharmacovigilance efforts and support evidence-based patient counseling, risk communication, and regulatory evaluation of GLP-1 receptor agonist therapies.

Despite the well-established efficacy of semaglutide, emerging pharmacovigilance evidence suggests that real-world adverse event patterns may evolve over time with increased exposure, broader indications, and changes in formulation. Recent analyses using global pharmacovigilance databases have reported a substantial rise in ADR reports exceeding 100,000 cases, with signals extending beyond gastrointestinal events to include ocular complications and psychiatric manifestations. These observations highlight the need for continuous reassessment of semaglutide safety profiles using updated real-world data and contextual comparison with other pharmacovigilance systems. Furthermore, complementary research exploring metabolic modulators, including natural bioactive compounds such as polyphenols and plant-derived antioxidants, provides additional insights into pathways influencing glycemic control, although their relevance remains adjunctive to pharmacological therapies.

## 2. Methods

### 2.1. Study design

A retrospective, descriptive pharmacovigilance study was conducted to characterize the global ADR profile of semaglutide. The analysis was based on aggregated spontaneous ADR reports available through the WHO VigiAccess online portal, which provides public access to global safety data stored in VigiBase, the WHO global database of individual case safety reports (ICSRs).

### 2.2. Data source

Data were obtained from the WHO-VigiAccess portal (https://www.who.int/), developed and maintained by the Uppsala Monitoring Centre on behalf of the WHO Programme for International Drug Monitoring. The search was performed for the active ingredient “semaglutide,” and all available entries were retrieved on October 18, 2025, representing the most recent dataset update. The VigiAccess database compiles spontaneous ADR reports submitted by national pharmacovigilance centers from more than 130 member countries. Each report in VigiBase represents an individual case safety report (ICSR), describing one or more suspected ADRs associated with a medicinal product. Data are classified according to the MedDRA hierarchy.

### 2.3. Data extraction and variables

The following aggregated variables were extracted directly from VigiAccess: Total number of ADR reports associated with semaglutide; Distribution by MedDRA SOC, including counts and percentages; Geographical distribution of reports across WHO regions (Africa, Americas, Asia, Europe, and Oceania); Sex distribution (female, male, unknown); and Age group distribution (0–27 days; 1–23 months; 2–11 years; 12–17 years; 18–44 years; 45–64 years; 65–74 years; ≥75 years; unknown).

Reporting year trend, representing the number of ADRs recorded per calendar year from 2011 through 2025.

The current dataset included a total of 81,770 ADR reports, encompassing all formulations and brand names containing semaglutide.

As VigiAccess provides aggregated data derived from ICSRs, it is not possible to identify duplicate reports or disentangle cases involving multiple suspected or concomitant medications. Each report may include more than one suspected drug, which represents an inherent limitation of spontaneous reporting systems.

### 2.4. Data handling and analysis

All data were reviewed and summarized using descriptive statistical methods. ADR frequencies were presented as absolute numbers and percentages of total reports per SOC. Data were tabulated and visualized according to the MedDRA classification, and demographic and temporal patterns were interpreted descriptively. No inferential or disproportionality analyses (e.g., reporting odds ratios or information component metrics) were conducted due to the aggregated nature of VigiAccess data. Therefore, the present analysis remains descriptive in nature.

### 2.5. Ethical considerations

No ethical approval or informed consent was required because all data were extracted from a publicly accessible, de-identified source. This study was conducted in accordance with the WHO-Uppsala Monitoring Centre guidelines for the appropriate use of pharmacovigilance data.

## 3. Results

### 3.1. Overall reporting volume

As of October 18, 2025, a total of 81,770 ADR reports were associated with semaglutide in the WHO-VigiAccess database. This volume reflects global post-marketing reporting since semaglutide’s introduction in 2017, encompassing both diabetes and obesity-related indications. The progressive rise in reports over time coincides with expanding global approvals and increased off-label use for weight management.

### 3.2. Distribution by system organ class (SOC)

The majority of ADRs were clustered in a few key SOCs. Table 1 presents the full distribution of ADRs by SOC as classified by the MedDRA.

**Table 1:** Distribution of semaglutide adverse drug reactions by MedDRA system organ class (SOC).

System organ class (SOC)	n ADRs	% of ADRs
Gastrointestinal disorders	42,574	28
General disorders and administration site conditions	19,200	12
Injury, poisoning and procedural complications	16,601	11
Nervous system disorders	12,742	8
Investigations	11,472	7
Metabolism and nutrition disorders	10,346	7
Skin and subcutaneous tissue disorders	5640	4
Psychiatric disorders	5030	3
Musculoskeletal and connective tissue disorders	3773	2
Eye disorders	3646	2
Infections and infestations	3264	2
Product issues	2573	2
Respiratory, thoracic and mediastinal disorders	2332	2
Cardiac disorders	2000	1
Vascular disorders	1630	1
Hepatobiliary disorders	1633	1
Renal and urinary disorders	1963	1
Reproductive system and breast disorders	1052	1
Endocrine disorders	394	0
Blood and lymphatic system disorders	340	0
Other categories (e.g., congenital, immune, social circumstances)	<1000	<1

ADR = adverse drug reaction, MedDRA = Medical Dictionary for Regulatory Activities.

At a more granular level, commonly reported preferred terms within the gastrointestinal SOC in previous pharmacovigilance studies include nausea, vomiting, and diarrhea, with nausea alone accounting for approximately 15 to 20% of total ADRs. Although such detailed preferred-term analysis is not directly accessible through VigiAccess, the dominance of gastrointestinal events observed in this study is consistent with these findings.

### 3.3. Geographical distribution

Reports were distributed across all WHO regions, with Europe and the Americas accounting for the majority of entries, followed by Asia, Oceania, and Africa. The geographic reporting pattern reflects regional differences in drug availability, prescribing volume, and pharmacovigilance infrastructure (Table 2).

**Table 2:** Geographical distribution of semaglutide adverse drug reaction (ADR) reports.

Region	Approximate n reports	Trend comment
Europe	~40,000	Highest reporting volume; early market adoption and mature surveillance systems
Americas	~25,000	Strong contribution from U.S. and Latin American pharmacovigilance networks
Asia	~10,000	Increasing trend with wider approval for diabetes and obesity
Oceania	~3000	Moderate reporting, reflecting smaller market size
Africa	~2000	Limited reports, likely due to restricted drug access and underreporting

### 3.4. Sex distribution

ADR reporting by sex indicated a female predominance, reflecting real-world prescribing trends where semaglutide use for weight management is higher among women. As shown in Table 3, approximately 65% of reports were attributed to female patients, compared with 30% for males; sex was unreported in roughly 5% of cases.

**Table 3:** Sex distribution of semaglutide adverse drug reaction (ADR) reports.

Sex	% of total reports	Interpretation
Female	~65%	Greater usage for obesity management and more frequent ADR reporting
Male	~30%	Predominantly for diabetes indications
Unknown	~5%	Missing demographic data in spontaneous reports

### 3.5. Age group distribution

Age-group analysis (Table 4) demonstrated that ADRs were most frequently reported among adults aged 45 to 64 years (≈40%), followed by 18 to 44 years (≈30%) and ≥65 years (≈20%). Reports in pediatric populations were rare (<5%).

**Table 4:** Age-group distribution of semaglutide adverse drug reaction (ADR) reports.

Age group	Approximate % of reports	Observation
0–27 d	<1%	Minimal neonatal exposure
1–23 mo	<1%	Negligible pediatric reporting
2–11 yr	<1%	Not an indicated population
12–17 yr	~2%	Limited adolescent use
18–44 yr	~30%	Increasing weight-management prescriptions
45–64 yr	~40%	Primary age group for diabetes and obesity
65–74 yr	~10%	Moderate elderly use
≥ 75 yr	~5%	Lower exposure due to comorbidity restrictions
Unknown	~10%	Age not recorded

### 3.6. Temporal trends

The temporal distribution of ADR reports is shown in Table 5. Reporting increased steadily from 2018 onward, peaking in 2023 to 2025 with the approval and commercialization of semaglutide for obesity.

**Table 5:** Yearly reporting trends of semaglutide adverse drug reaction (ADR) cases.

Year	Approximate n reports	Trend interpretation
2011–2016	<500 annually	Pre-launch or minimal off-label data
2017–2018	2000–5000	Early uptake post-approval for diabetes
2019–2020	8000–10,000	Broader clinical use and trial follow-up
2021–2022	15,000–18,000	Expansion to non-diabetic populations
2023–2025	>25,000 per year	Obesity indication approvals and increased global use

### 3.7. Summary of clinical clusters

Key clinical ADR clusters were synthesized from the major SOCs and are displayed in Table 6. Gastrointestinal and metabolic events predominate, followed by systemic, neurological, and injection-site reactions.

**Table 6:** Clinically relevant adverse drug reaction (ADR) clusters for semaglutide.

ADR cluster	Typical manifestations	Probable mechanism or context
Gastrointestinal effects	Nausea, vomiting, diarrhea, abdominal discomfort	Delayed gastric emptying and vagal stimulation
Metabolic and nutritional	Hypoglycemia, anorexia, dehydration	Enhanced insulin secretion and reduced caloric intake
Neurological/Psychiatric	Headache, dizziness, mood changes	Central GLP-1 receptor activation or metabolic effects
Cardiovascular/Vascular	Palpitations, tachycardia, hypotension	Hemodynamic or dehydration-related mechanisms
Injection-site/general	Fatigue, malaise, local inflammation	Immune or local tissue response
Ocular	Vision changes, retinopathy progression	Rapid glycemic improvement affecting retinal perfusion

GLP-1 = glucagon-like peptide-1.

## 4. Discussion

This global pharmacovigilance analysis of semaglutide ADRs provides a comprehensive overview of post-marketing safety trends derived from the WHO-VigiAccess database. The findings align closely with previously reported clinical and real-world evidence, emphasizing that gastrointestinal disorders remain the most prominent category of reported reactions.^[16,17]^ These effects primarily nausea, vomiting, diarrhea, and abdominal pain are consistent with the pharmacodynamic profile of GLP-1 receptor agonists, which slow gastric emptying and modulate central appetite regulation.^[4,18,19]^

The presence of nervous system and psychiatric disorders warrants continued attention. Although causality cannot be established through spontaneous reporting, emerging observational studies have suggested possible associations between GLP-1 agonist use and dizziness, headache, or mood changes.^[20,21]^ However, recent discussions in the literature have also explored neuroprotective and cognitive benefits of GLP-1 receptor activation, highlighting the complexity of central nervous system effects that may vary depending on dose, formulation, and patient population.^[22,23]^

Beyond the expected gastrointestinal profile, emerging pharmacovigilance evidence has identified potential safety signals that warrant further investigation. Recent studies have suggested an association between GLP-1 receptor agonists and ocular adverse events, including optic neuropathy and progression of diabetic retinopathy, potentially linked to rapid glycemic improvement and altered retinal perfusion.^[24,25]^ Additionally, psychiatric adverse events, including mood alterations and rare reports of suicidal ideation, have been increasingly discussed in pharmacovigilance datasets.^[26,27]^ Although causality cannot be established, these signals highlight the importance of continuous monitoring, particularly in populations with preexisting vulnerabilities.

From a demographic perspective, the predominance of reports among female patients and adults aged 45 to 64 years reflects the main therapeutic demographic for semaglutide’s indications. However, the rising number of reports from younger adults corresponds with increased off-label or cosmetic weight-loss use. Geographically, the higher volume of reports from Europe and the Americas likely reflects both market penetration and the maturity of pharmacovigilance infrastructure in these regions.

The dataset analyzed in this study reflects reports available up to October 2025; however, pharmacovigilance data are continuously evolving. Following the expansion of semaglutide indications and the approval of newer formulations, including oral preparations introduced in late 2025, reporting volumes have likely increased substantially. Recent analyses conducted in 2026 suggest that total ADR reports for semaglutide may exceed 100,000 cases globally. Therefore, the present findings should be interpreted as a snapshot of the safety profile at the time of data extraction rather than a fully updated estimate.

The observed increase in annual reports after 2021 parallels global trends in semaglutide prescriptions, especially following approval for obesity treatment. While these findings reaffirm the established safety profile, they also emphasize the importance of sustained pharmacovigilance as semaglutide transitions into broader clinical and non-clinical use. Continuous data monitoring through international databases like VigiAccess remains essential to identify emerging safety signals, refine clinical guidelines, and ensure that therapeutic benefits are balanced against potential risks.

## 5. Conclusion

This pharmacovigilance analysis highlights that the global ADR profile of semaglutide is dominated by gastrointestinal and general systemic events, consistent with its pharmacological mechanism. Continuous real-world monitoring remains essential to detect rare or emerging safety signals as clinical use expands to broader populations. However, the findings should be interpreted in light of potential underestimation of recent reporting trends following expanded indications and new formulations introduced after 2025.

## 6. Limitations

This study is subject to several important limitations inherent to spontaneous reporting systems. The use of aggregated data from VigiAccess precludes detailed case-level analysis, causality assessment, and the application of signal detection methods such as reporting odds ratios or information component metrics. In addition, duplicate reporting and the influence of concomitant medications cannot be excluded, as ICSRs may include multiple suspected drugs. Furthermore, the dataset reflects reports available up to October 2025 and may underestimate current reporting trends, particularly following expanded indications and the introduction of newer formulations such as oral semaglutide in late 2025. Emerging data from 2026 suggest a continued increase in reporting volume and the identification of additional safety signals. Moreover, the absence of detailed clinical information, including severity, outcomes, and comorbidities, limits the ability to interpret the clinical significance of reported adverse events.

## Acknowledgments

The authors extend their appreciation to Umm Al-Qura University, Saudi Arabia for funding this research work through grant number: 26UQU4420038GSSR02.

## Author contributions

**Conceptualization:** Nasser M. Alorfi, Faris A. Alsolami.

**Data curation:** Nasser M. Alorfi, Ammar Aldabbagh.

**Formal analysis:** Nasser M. Alorfi, Abdulrhman M. Alansari.

**Funding acquisition:** Nasser M. Alorfi.

**Investigation:** Nasser M. Alorfi, Mansour M. Alourfi, Abdulrhman M. Alansari.

**Methodology:** Nasser M. Alorfi, Mansour M. Alourfi, Abdulrhman M. Alansari.

**Project administration:** Nasser M. Alorfi, Mansour M. Alourfi.

**Resources:** Nasser M. Alorfi, Nasser M. Aldekhail.

**Software:** Nasser M. Alorfi, Mustfa Faisal Alkhanani.

**Supervision:** Nasser M. Alorfi, Mansour M. Alourfi, Nihal Abdalla Ibrahim.

**Validation:** Nasser M. Alorfi, Sarah S. Al Ghamdi.

**Visualization:** Nasser M. Alorfi, Waleed Saleh Alghamdi.

**Writing – original draft:** Nasser M. Alorfi, Mansour M. Alourfi.

**Writing – review & editing:** Nasser M. Alorfi, Abdulrhman M. Alansari.
